# Evaluating the role of serum IL1R2 as a biomarker for diagnosis and prognostic stratification in sepsis

**DOI:** 10.1186/s40001-026-03939-3

**Published:** 2026-02-01

**Authors:** Yusheng Wang, Yuxian Wu, Zeping Jiang, Qian Lin, Min Wu, Jiansui Xu, Ting Sun, Meitang Wang, Yaoyang Liu, Yang Liu

**Affiliations:** 1 Department of Critical Care Medicine, Chinese People’s Liberation Army Naval Medical Center, Shanghai, China; 2 Department of Emergency, Changhai Hospital, Naval Medical University, Shanghai, China; 3 Department of Biological Therapy, The Third Affiliated Hospital, Naval Medical University, Shanghai, China; 4 Department of Rehabilitation Medicine, Shanghai Fourth People’s Hospital affiliated to Tongji University School of Medicine, Shanghai, China

**Keywords:** IL1R2, Sepsis, Biomarker, Diagnosis, Prognosis

## Abstract

**Objective:**

This study aimed to identify novel sepsis biomarkers by evaluating serum interleukin-1 receptor type 2 (IL1R2) for its diagnostic and prognostic utility, in light of the limitations of current markers like PCT and CRP.

**Methods:**

A single-center retrospective analysis was conducted involving 55 sepsis patients and 42 non-sepsis controls. Serum IL1R2 levels, measured via ELISA within 24 h of admission, were compared against clinical data, including SOFA scores, 28-day mortality, and laboratory parameters (PCT, CRP). Diagnostic performance was assessed using ROC curve analysis, while prognostic utility was determined via Kaplan–Meier analysis. A cecal ligation and puncture (CLP) was used to track IL1R2 dynamics over time.

**Results:**

Sepsis patients exhibited significantly elevated serum IL1R2 levels compared to controls. IL1R2 demonstrated strong diagnostic power (AUC = 0.908), outperforming PCT and CRP. Furthermore, higher IL1R2 levels correlated with increased SOFA scores and predicted poorer 28-day survival. In the CLP model, serum IL1R2 rose within 4 h post-sepsis, peaked within 24 h, returned to baseline by day 3, and fell below normal by day 7.

**Conclusion:**

Serum IL1R2 is a promising biomarker, offering a superior ability to correlate with disease severity and predict 28-day mortality.

## Introduction

Sepsis is a life-threatening organ dysfunction caused by a dysregulated host immune response to infection [1]. According to the Global Burden of Disease Report, sepsis-related deaths account for nearly 20% of all global fatalities [2]. Current diagnosis of sepsis primarily relies on clinical assessment and conventional infection biomarkers such as procalcitonin (PCT) and C-reactive protein (CRP) [3]. However, their utility is limited by variability in individual responses and the influence of non-infectious factors: PCT can also be elevated in non-infectious inflammatory conditions [4], while CRP exhibits a broad and delayed response, failing to accurately reflect the transition in immune status specific to sepsis or the risk of disease progression [5]. Consequently, there is a pressing need for novel biomarkers that can precisely identify septic immune endotypes, assess organ injury, and predict clinical outcomes to facilitate early intervention and personalized therapy.

During infection, the interleukin-1 (IL-1) system plays a central role in initiating inflammatory responses and regulating antimicrobial immunity [2, 6]. Interleukin-1 receptor 2 (IL1R2), a decoy receptor for IL-1, structurally resembles the functional receptor IL-1R1 in its extracellular domain and competitively binds IL-1α and IL-1β [7]. However, due to the absence of an intracellular TIR domain, it cannot initiate downstream pro-inflammatory signaling, thereby exhibiting significant anti-inflammatory properties [8]. IL1R2 is primarily expressed on myeloid cells such as macrophages, neutrophils, and certain T cells [9]. Its expression is upregulated in various pathological states-including infectious diseases, inflammatory bowel diseases and cancer-and is closely associated with disease severity [10–12]. Recent studies have further revealed that IL1R2 participates in immune regulation through multiple mechanisms, such as promoting tumor cell proliferation [11], inducing T cell exhaustion [13], and regulating macrophage polarization toward the M2 phenotype [14].

Notably, emerging clinical evidence indicates that soluble IL1R2 (sIL1R2) is significantly elevated in the serum of sepsis patients [12]. Moreover, under septic conditions, IL1R2 expression exhibits cell type specificity, with particularly prominent upregulation in monocytes, suggesting a potential key role in monocyte-mediated immunosuppression [15]. These findings highlight the importance of IL1R2 as a potential biomarker in sepsis. However, there is a paucity of research focusing on the biomarker potential of serum IL1R2 in sepsis.

Therefore, this study aims to comprehensively validate the clinical value of serum IL1R2 in sepsis by firstly determining its diagnostic sensitivity and specificity, and secondly, investigating its capacity to indicate disease severity and predict patient prognosis.

## Method

### Patient enrollment

A retrospective analysis was conducted on 97 adult patients admitted to the ICU of the Naval Medical Center of People’s Liberation Army from March 2024 to May 2025. Sepsis patients were defined per Sepsis-3 criteria, while the non-sepsis control group comprised patients with trauma, coronary heart disease, post-surgery status, or infection (SOFA < 2). Collected data included demographics, comorbidities, infection details, SOFA, hospital stay duration, 28-day mortality, and laboratory results.

### Sample collection and IL1R2 measurement

Blood samples were collected within 24 h after admission. The blood samples were allowed to clot for 1 h at room temperature before centrifugation at 3000 × g for 15 min. The serum was then transferred to sterile tubes and stored at − 80 °C for up to six months prior to IL1R2 measurement. The concentration of serum IL1R2 was detected using enzyme-linked immunosorbent assay (ELISA) in accordance with the manufacturer's protocol (RayBio^®^ Human IL-1 R2 ELISA Kit, Catalog #: ELH-IL1R2).

### Mouse model of sepsis

8-week-old male C57 mice (Shanghai Sippe-BK Laboratory Animal Co. Ltd)) were used to establish sepsis model via cecal ligation and puncture (CLP) [16]. 4–5% isoflurane was used by inhalation to induce anaesthesia prior to surgery, with 1–2% isoflurane administered during the procedure to maintain anaesthesia. For euthanasia, mice were first anaesthetised with 4–5% isoflurane inhalation before undergoing cervical dislocation. Serum IL-1R2 levels were measured by ELISA according to manufacturer's protocol (RayBio^®^ Mouse IL-1 R2 ELISA Kit, Catalog #: ELM-IL1R2) at 2, 6, 12, 24 h, 3 days, and 7 days post-CLP via blood sampling from the inferior vena cava, these time points were chosen to capture the early hyperinflammatory phase, the subsequent immunosuppressive phase, and the late resolution phase typical of the murine CLP model.

### Statistical analysis

Quantitative data are presented as mean ± standard deviation (SD) or median (interquartile range). Categorical data are expressed as percentages. The Shapiro–Wilk test was used to assess whether variables of interest followed a normal distribution. Comparisons between groups with normally distributed data were performed using the t-test or one-way analysis of variance (ANOVA), while non-normally distributed data were analyzed using nonparametric tests or the two-tailed Mann–Whitney U test. Correlations were evaluated using Pearson correlation (Normally distributed data) or Spearman correlation (Non-normal distribution data) coefficient along with corresponding p-values. To assess the sensitivity and specificity of IL-1R2, PCT, and CRP expression as biomarkers in non-sepsis, sepsis, and septic shock patient cohorts, receiver operating characteristic (ROC) curve analysis was conducted, and the area under the curve (AUC) was calculated. The optimal cutoff value is determined by the Youden index.

## Results

### Patient characteristics

This retrospective analysis included 55 sepsis patients and 42 non-sepsis controls. Baseline characteristics are summarized in Table 1. The sepsis cohort consisted predominantly of elderly males, with pulmonary and abdominal infections being the most prevalent. Gram-negative bacteria were the primary causative pathogens. Shock developed in 76.4% of sepsis patients, and 60.0% required mechanical ventilation. Over the 28-day follow-up period, the all-cause mortality rate was 49.1%.

**Table 1:** Baseline characteristics of non-sepsis and sepsis patients enrolled

Characteristics	Sepsis	Non-sepsis	*p* value
n	55	42	
Age, median (IQR)	73.0 (64.0, 78.0)	66.5 (57.3, 76.0)	0.088
Gender, n (%)			0.004
Male	40 (74.1%)	19 (45.2%)	
Female	14 (25.9%)	23 (54.8%)	
Comorbidities, n (%)			
Hypertension, n (%)	22 (40%)	14 (33.3%)	0.501
Diabetes mellitus, n (%)	10 (18.2%)	10 (23.8%)	0.497
Coronary artery disease, n (%)	9 (16.4%)	1 (2.4%)	0.057
Liver disease, n (%)	2 (3.6%)	2 (4.8%)	1
Kidney disease, n (%)	8 (14.5%)	0 (0%)	0.027
Malignancy, n (%)	17 (30.9%)	1 (2.4%)	< 0.001
Central nervous disease, n (%)	3 (5.5%)	2 (4.8%)	1
Infection site, n (%)			< 0.001
Lung	21 (38.2%)	7 (16.7%)	
Abdomen	19 (34.5%)	0 (0%)	
Blood	4 (7.3%)	0 (0%)	
Soft tissue	2 (3.6%)	0 (0%)	
Genitourinary	2 (3.6%)	3 (7.1%)	
Others	7(12.7%)	0 (0%)	
Pathogen, n (%)			< 0.001
G-	35 (63.6%)	8 (20%)	
G +	12 (21.8%)	0 (0%)	
Unclear	6 (10.9%)	0 (0%)	
Fungi	2 (3.6%)	0 (0%)	
Severity, treatment and survival			
Shock, n (%)	42 (76.4%)	0 (0%)	< 0.001
Ventilator	33 (60%)	0 (0%)	< 0.001
CRRT, n (%)	11 (20%)	0 (0%)	0.009
28-day mortality, n (%)	27 (49.1%)	2 (4.8%)	< 0.001
Survival time, median (IQR)	14 (5.5, 28)	26 (10, 28)	0.04
SOFA, median (IQR)	9.5 (7, 14)	0 (0, 1)	< 0.001
Sepsis biomarkers			
Lactate, median (IQR)	2.55 (1.68, 4.30)	1.15 (0.83, 1.40)	< 0.001
PCT, median (IQR)	9.93 (2.07, 33.33)	0.31 (0.09, 0.60)	< 0.001
IL6, median (IQR)	304.60 (56.35, 837.80)	38.10 (18.90, 64.40)	< 0.001
IL-1R2, median (IQR)	65.33 (37.89, 133.13)	25.85 (23.11, 32.02)	< 0.001
CRP, median (IQR)	116.00 (75.25, 194)	35.70 (17.75, 56.05)	< 0.001
Laboratory results			
BNP, median (IQR)	823.10 (283.40, 1825.80)	83.35 (41.85, 146.73)	< 0.001
Troponin, median (IQR)	0.09 (0.04, 0.83)	0.006 (0.004, 0.008)	< 0.001
ALT, median (IQR)	42.00 (21.95, 82.95)	23.50 (16.18, 32.75)	0.016
AST, median (IQR)	66.65 (30.78, 160.25)	23.35 (17.85, 39.50)	< 0.001
Bilirubin, median (IQR)	22.85 (15.68, 50.20)	13.65 (9.73, 18.45)	< 0.001
Albumin, mean ± sd	31.43 ± 4.73	38.21 ± 5.48	< 0.001
Creatinine, median (IQR)	124.00 (87.25, 291.50)	52.00 (47.00, 68.00)	< 0.001
PT, median (IQR)	16.65 (14.28, 19.40)	13.10 (12.33, 14.68)	< 0.001
INR, median (IQR)	1.37 (1.18, 1.66)	1.07 (0.99, 1.14)	< 0.001
APTT, mean ± sd	45.2 0± 14.51	34.46 ± 6.08	< 0.001
TT, median (IQR)	17.10 (16.08, 19.23)	16.00 (15.00, 16.70)	< 0.001
Fibrinogen, median (IQR)	4.03 (2.46, 5.66)	3.46 (2.97, 4.40)	0.378
D-dimer, median (IQR)	4.56 (2.62, 9.20)	0.93 (0.60, 2.24)	< 0.001
WBC, median (IQR)	11.05 (6.47, 15.89)	8.40 (6.72, 11.94)	0.212
Hb, mean ± sd	86.76 ± 25.68	102.51 ± 19.48	0.002
PLT, median (IQR)	106.50 (52.50, 171.25)	205.00 (156.00, 308.50)	< 0.001
N%, median (IQR)	88.00 (85.00, 91.70)	81.00 (71.70, 86.15)	< 0.001
L%, median (IQR)	4.70 (3.23, 8.23)	10.60 (6.05, 16.25)	< 0.001

Normally distributed data were presented as mean ± standard deviation (SD), non-normal data as median (interquartile range, IQR). *ALT* Alanine Aminotransferase, *APTT* Activated Partial Thromboplastin Time; *AST* Aspartate Aminotransferase, *CRP* C-Reactive Protein, *CRRT* Continuous Renal Replacement Therapy, *INR* International Normalized Ratio; *L%* Lymphocyte Percentage; *N%* Neutrophil Percentage; *PCT* Procalcitonin; *PLT* Platelet Count; *PT* Prothrombin Time; *SOFA* Sequential Organ Failure Assessment; *TT* Thrombin Time; *WBC* White Blood Cell Count

### Serum IL1R2 levels significantly elevated in sepsis patients

**Fig. 1:**
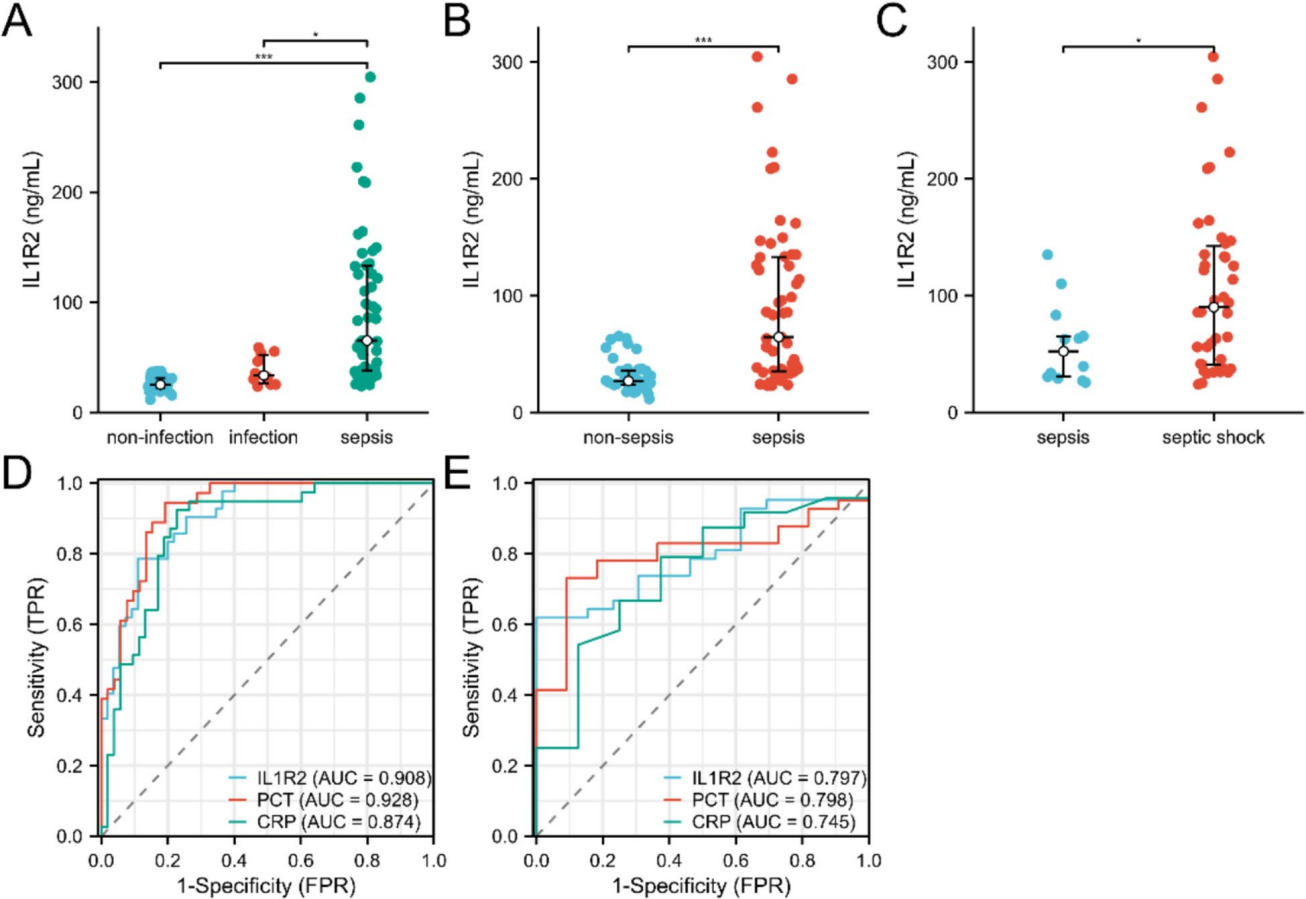
Serum IL1R2 level elevated in sepsis patients. Serum IL1R2 level significantly elevated in non-infection (n = 32) vs infection (n = 10) vs sepsis (n = 55, **A**), non-sepsis (n = 42) vs sepsis (n = 55, **B**), and sepsis (n = 13) vs septic shock (n = 42, **C**) patients. ROC analysis for IL1R2, PCT, CRP in distinguishing non-sepsis and sepsis (**D**), sepsis and septic shock (**E**). *p < 0.05, ***p < 0.001

As shown in Fig. 1, compared with non-infected patients, serum IL1R2 levels were elevated in infected patients but did not reach statistical significance, whereas they were significantly elevated in sepsis patients (Fig. 1A, B). Concurrently, serum IL1R2 levels were further elevated in septic shock patients (Fig. 1C). The ROC curve analysis revealed that serum IL1R2 yielded an AUC of 0.908 for sepsis diagnosis, which was slightly lower than that of PCT (AUC = 0.928) but higher than that of CRP (AUC = 0.874) (Fig. 1D). In terms of distinguishing sepsis patients from those with septic shock, serum IL-1R2 demonstrated comparable diagnostic performance to PCT (AUC = 0.797 vs 0.798) and superior performance to CRP (AUC = 0.745) (Fig. 1E). Its sensitivity was 0.79, marginally lower than both PCT and CRP, yet its specificity reached 0.89, exceeding that of both PCT and CRP. Table 2 illustrated diagnostic performance of IL1R2, PCT, and CRP.

**Table 2:** Diagnostic performance of IL1R2, PCT and CRP

Biomarker	Cut-off	Sensitivity	Specificity	Accuracy	Positive predictive value	Negative predictive value	Youden’s index
IL1R2	32.994	0.78571	0.89091	0.84536	0.84615	0.84483	0.67662
PCT	1.495	0.94444	0.80769	0.86364	0.77273	0.95455	0.75214
CRP	71.185	0.92308	0.77358	0.83696	0.75000	0.93182	0.69666

*IL1R2* Interleukin-1 receptor type 2, *PCT* Procalcitonin, *CRP* C-Reactive Protein

### Positive correlation between serum IL1R2 and sepsis severity

Patients were grouped as IL1R2-high or IL1R2-low based on median serum IL1R2 levels in sepsis patients. As shown in Table 3, no significant differences were observed between groups in age, comorbidities, or site of infection. The difference in sex ratio may stem from bias arising from the underrepresentation of female patients in the sepsis cohort. Regarding pathogen types, patients with elevated IL1R2 levels exhibited a higher proportion of Gram-negative infections. Concurrently, the IL1R2 high group demonstrated significantly higher rates of shock occurrence, mechanical ventilation, SOFA scores, and 28-day mortality. Regarding pathogen types, patients with elevated IL1R2 levels exhibited a higher proportion of Gram-negative infections. Concurrently, the IL1R2 high group demonstrated significantly higher rates of shock occurrence, mechanical ventilation, SOFA scores, and 28-day mortality. Laboratory assessments including hepatic and renal function, coagulation parameters, and complete blood counts further indicated more severe disease severity in patients with elevated IL1R2 levels.

**Table 3:** Comparison of baseline characteristics of sepsis patients grouped according to median IL1R2 level

Characteristics	Low	High	*p* value
n	28	27	
Age, median (IQR)	69 (60, 78)	73 (64.5, 78)	0.472
Gender, n (%)			0.013
Male	24 (88.9%)	16 (59.3%)	
Female	3 (11.1%)	11 (40.7%)	
Comorbidities, n (%)			
Hypertension, n (%)	11 (39.3%)	11 (40.7%)	0.912
Diabetes mellitus, n (%)	3 (10.7%)	7 (25.9%)	0.266
Coronary artery disease, n (%)	4 (14.3%)	5 (18.5%)	0.952
Liver disease, n (%)	1 (3.6%)	1 (3.7%)	1.000
Kidney disease, n (%)	5 (17.9%)	3 (11.1%)	0.744
Malignancy, n (%)	11 (39.3%)	6 (22.2%)	0.171
Central nervous disease, n (%)	3 (10.7%)	0 (0%)	0.248
Infection site, n (%)			0.404
Lung	10 (35.7%)	11 (40.7%)	
Abdomen	8 (28.6%)	11 (40.7%)	
Blood	2 (7.1%)	2 (7.4%)	
Soft tissue	1 (3.6%)	1 (3.7%)	
Genitourinary	2 (7.1%)	0 (0%)	
Others	5 (17.9%)	2 (7.4%)	
Pathogen, n (%)			0.029
G-	14 (50%)	21 (77.8%)	
Fungi	9 (32.1%)	3 (11.1%)	
G +	0 (0%)	2 (7.4%)	
Unclear	5 (17.9%)	1 (3.7%)	
Severity, treatment and survival			
SOFA, mean ± sd	7.30 ± 4.20	12.20± 4.00	< 0.001
Shock, n (%)	16 (57.1%)	26 (96.3%)	< 0.001
ventilator, n (%)	11 (39.3%)	22 (81.5%)	0.001
CRRT, n (%)	4 (14.3%)	7 (25.9%)	0.281
28-day mortality, n (%)	7 (25%)	20 (74.1%)	< 0.001
Survival time, median (IQR)	28.00 (26.75, 28.00)	8.00 (4.50, 23.50)	< 0.001
Sepsis biomarkers			
Lactate, median (IQR)	2.30 (1.23, 3.75)	3.30 (2.20, 6.05)	0.054
PCT, median (IQR)	5.64 (0.91, 17.68)	12.26 (5.82, 37.83)	0.071
CRP, mean ± sd	122.90 ± 61.04	134.08 ± 46.50	0.581
IL6, median (IQR)	85.09 (31.03, 180.45)	495.70 (424.20, 1566.00)	0.009
Laboratory results			
BNP, median (IQR)	508.80(269.80, 1156.50)	1466.20 (433.02, 2894.40)	0.278
Troponin, median (IQR)	0.06 (0.02, 0.75)	0.14 (0.07, 1.24)	0.155
ALT, median (IQR)	30.40 (15.50, 54.68)	47.25 (27.78, 111.50)	0.077
AST, median (IQR)	45.90 (20.88, 81.38)	84.95 (47.15, 442.03)	0.006
Bilirubin, median (IQR)	20.50 (13.95, 30.93)	29.95 (17.20, 87.63)	0.061
Albumin, mean ± sd	32.42 ± 4.45	30.44 ± 4.89	0.140
Creatinine, median (IQR)	102.90 (72.73, 274.00)	134.50 (110.50, 276.27)	0.224
PT, median (IQR)	16.15 ± 3.17	18.66 ± 4.64	0.027
APTT, median (IQR)	41.56 ± 11.70	48.84 ± 16.27	0.070
TT, median (IQR)	17.25 (16.15, 18.93)	17.00 (16.03, 19.35)	0.913
Fibrinogen, median (IQR)	4.04 (2.63, 5.01)	3.92 (1.98, 5.97)	0.869
D-dimer, median (IQR)	3.93 (2.23, 5.69)	6.00 (2.94, 11.75)	0.076
WBC, mean ± sd	10.42 ± 5.60	12.89 ± 7.04	0.163
Hb, mean ± sd	82.76 ± 24.98	90.92 ± 26.21	0.251
PLT, median (IQR)	142.50 (72.75, 228.25)	78.00 (45.75, 143.00)	0.031
N%, median (IQR)	86.00 (81.70, 89.35)	91.25 (86.58, 93.45)	0.003
L%, median (IQR)	4.70 (3.70, 9.20)	4.20 (2.40, 7.40)	0.252

Normally distributed data were presented as mean ± standard deviation (SD), non-normal data as median (interquartile range, IQR). *ALT* Alanine Aminotransferase, *APTT* Activated Partial Thromboplastin Time, *AST* Aspartate Aminotransferase, *CRP* C-Reactive Protein, *CRRT* Continuous Renal Replacement Therapy, *INR* International Normalized Ratio, *L%* Lymphocyte Percentage, *N%* Neutrophil Percentage, *PCT* Procalcitonin, *PLT* Platelet Count, *PT* Prothrombin Time, *SOFA* Sequential Organ Failure Assessment, *TT* Thrombin Time, *WBC* White Blood Cell Count

**Fig. 2:**
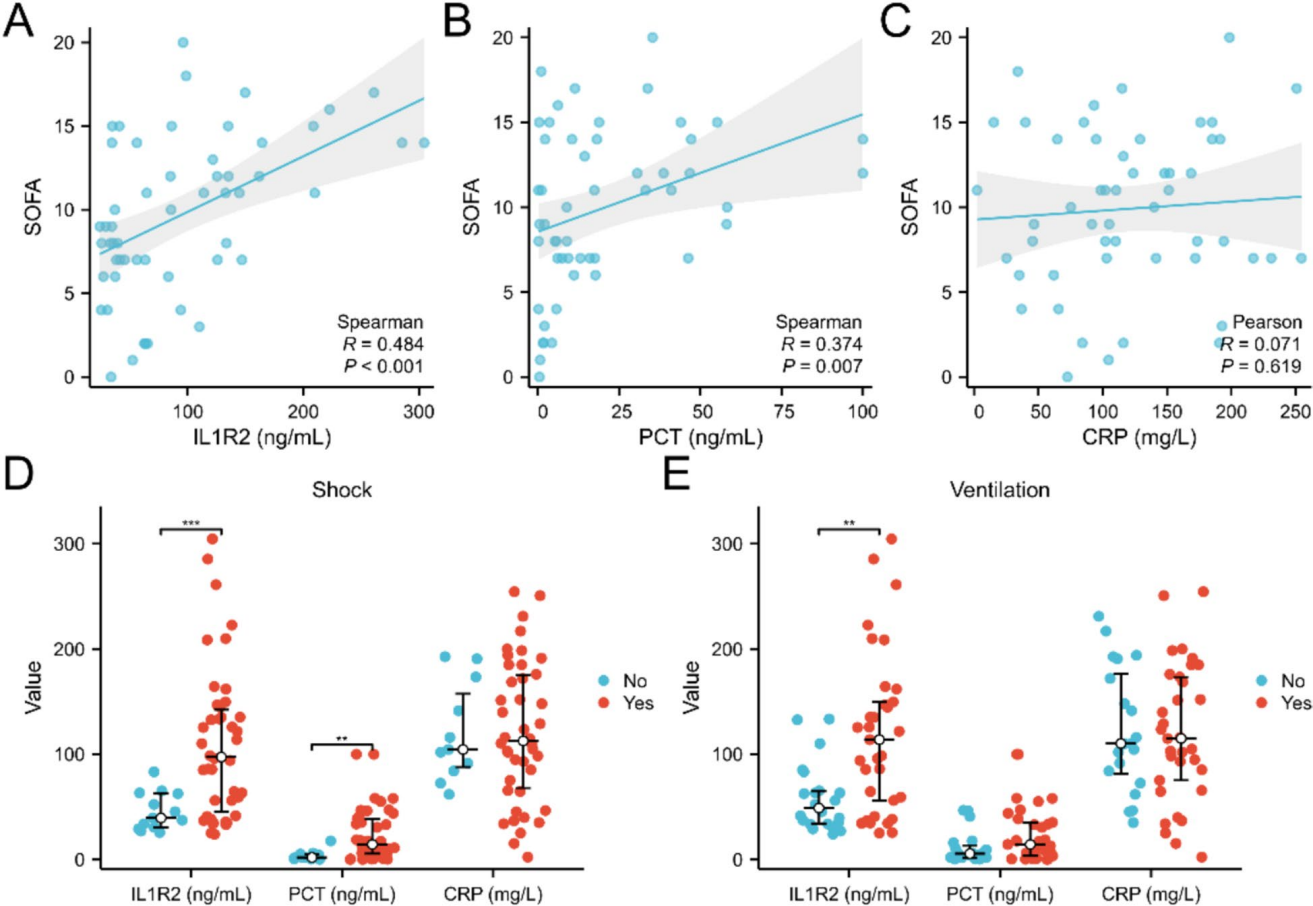
Serum IL1R2 positively correlated with sepsis severity. Correlation analysis of SOFA and serum IL1R2 (**A**, n = 54), PCT (**B**, n = 51), and CRP (**C**, n = 52). Serum IL1R2 (n = 55), PCT (n = 52), and CRP (n = 53) levels in sepsis patients with or without shock (**D**), and mechanical ventilator use (**E**). **p < 0.01, ***p < 0.001

Correlation analysis revealed a significantly positive association between serum IL1R2 levels and SOFA scores (R = 0.484, *p* < 0.001, Fig. 2A). PCT also demonstrated significant correlation with SOFA scores, but with a lower correlation coefficient (R = 0.374, *p* = 0.007, Fig. 2B). CRP showed no correlation with SOFA scores (Fig. 2C). When patients were grouped according to shock status and mechanical ventilation use, results indicated that IL1R2 and PCT levels were significantly higher in patients with shock compared to those without shock, while CRP levels showed no significant difference (Fig. 2D). Among patients receiving mechanical ventilation, IL1R2 levels were significantly higher than in those not receiving ventilation, with no significant differences in PCT or CRP levels (Fig. 2E).

### Predictive value of serum IL1R2 for 28-day mortality

**Fig. 3:**
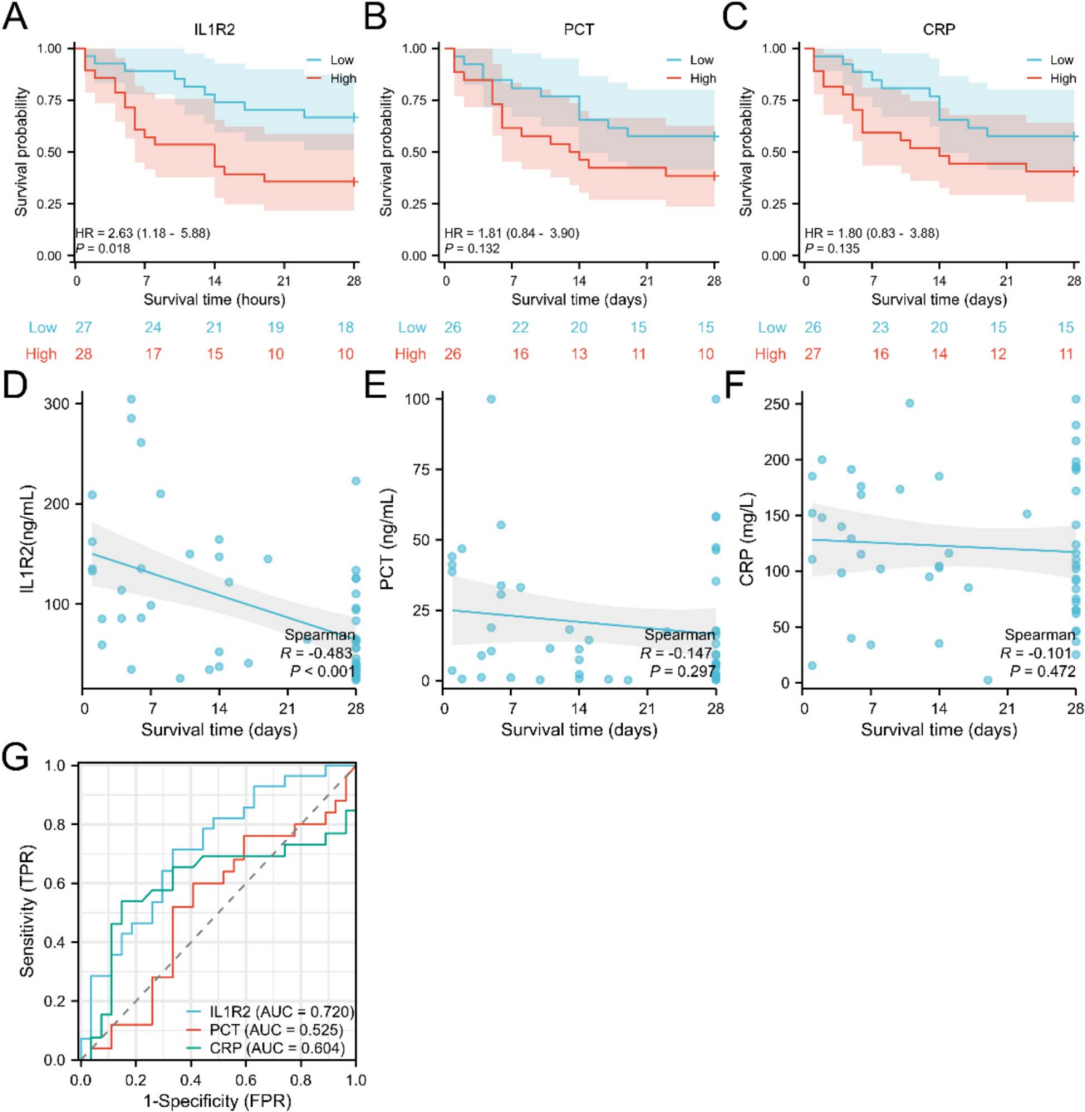
Prognostic utility of IL1R2 and PCT, CRP. KM analysis of IL1R2 (**A**, n = 55), PCT (**B**, n = 52), CRP (**C**, n = 53). Correlation analysis of survival time and IL1R2 (**D**, n = 55), PCT (**E**, n = 52), CRP (**F**, n = 53). ROC analysis for IL1R2, PCT, CRP in differentiating 28-day outcome (**G**)

Based on median serum IL1R2 levels, KM analysis revealed significantly higher 28-day mortality in patients with elevated serum IL1R2 level (Fig. 3A, HR = 2.63, *p* = 0.018). Patients with elevated PCT and CRP levels also exhibited a tendency towards increased mortality, though statistical significance was not attained (Fig. 3B, C). Correlation analysis demonstrated a significant negative association between serum IL1R2 levels and survival time (Fig. 3D, R = −0.483, *p* < 0.001), whereas neither PCT nor CRP correlated with survival time (Fig. 3E, F). ROC analysis (Fig. 3G) demonstrated that serum IL1R2 achieved an AUC of 0.720 for predicting 28-day survival outcomes, surpassing both PCT (AUC = 0.525) and CRP (AUC = 0.604). Cox regression analysis showed that IL1R2 is an independent risk factor for 28-day mortality in sepsis patient (Table 4).

**Table 4:** Univariate and multivariate Cox regression model of risk factor predicting 28-day mortality

Characteristics	Total(N)	Univariate analysis		Multivariate analysis	
		Hazard ratio (95% CI)	P value	Hazard ratio (95% CI)	*p* value
IL1R2	55	1.008 (1.003–1.013)	0.001	1.007 (1.001—1.012)	0.012
Lactate	48	1.126 (0.970–1.306)	0.118		
PCT	52	1.004 (0.990–1.019)	0.546		
CRP	53	1.003 (0.998–1.009)	0.206		
Bilirubin	52	0.994 (0.985–1.003)	0.190		
Creatinine	52	1.000 (0.999–1.002)	0.660		
D-dimer	52	0.994 (0.930–1.063)	0.871		
WBC	53	0.990 (0.934–1.050)	0.745		
Hb	53	1.016 (1.000–1.033)	0.049	1.010 (0.993—1.026)	0.247
PLT	52	0.999 (0.996–1.002)	0.474		

*CRP* C-Reactive Protein, *Hb* hemoglobin, *PCT* Procalcitonin, *PLT* Platelet Count, *WBC* White Blood Cell Count

### Kinetics of serum IL1R2 in mouse sepsis model

**Fig. 4:**
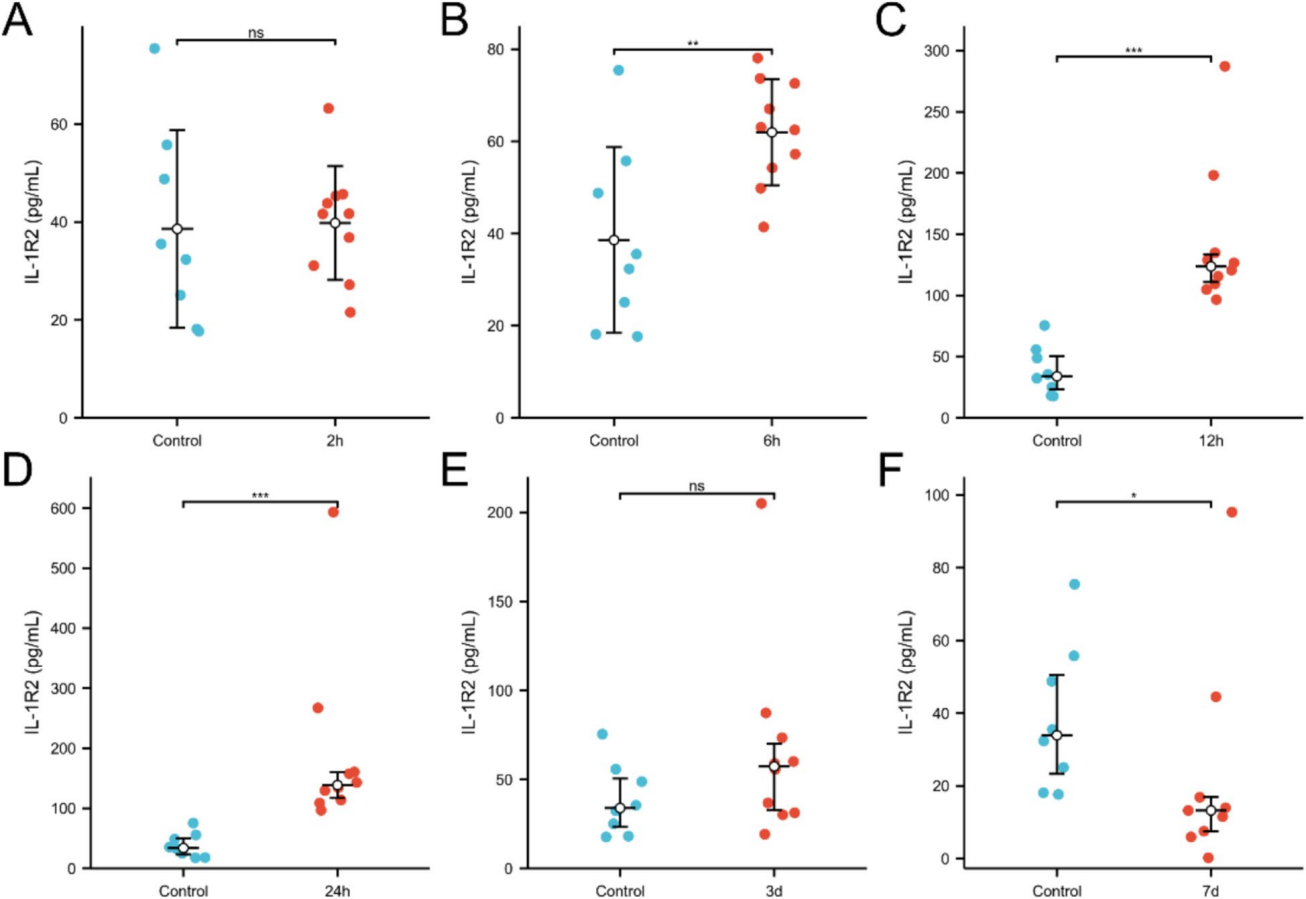
Kinetics of serum IL1R2 in mouse CLP model. Comparison of control (n = 10) and serum IL1R2 level in 2h (**A**, n = 10), 6h (**B**, n = 10), 12h (**C**, n = 10), 24h (**D**, n = 10), 3 day (**E**, n = 10), 7 day (**F**, n = 9) post-CLP. *p < 0.05, **p < 0.01, ***p < 0.001. ns, no significance

To further investigate the pattern of elevated serum IL1R2 in sepsis, a mouse sepsis model was established via CLP, and measured serum IL1R2 levels at various time points post-modelling. Results demonstrated that serum IL1R2 levels were not significantly elevated at 2h post-CLP (Fig. 4A). The earliest significant increase occurred at 6h post-CLP (Fig. 4B), with continued elevation observed at 12 and 24h (Figs. 4C, D). By day 3 post-CLP, serum IL1R2 levels had returned to control group levels (Fig. 4E), whereas by day 7 levels had further decreased to levels significantly lower than the control group (Fig. 4F).

## Discussion

Sepsis represents a significant global public health burden, with mortality rates ranging from 25 to 50%, accounting for approximately 20% of all causes of death annually [17]. The absence of specific early manifestations in sepsis renders clinical diagnosis challenging, whereas biomarkers offer objective indicators to enhance diagnostic accuracy. Furthermore, an ideal biomarker should possess multiple functions, including reflecting the magnitude of organ dysfunction, facilitating risk stratification, and enabling prognostic assessment [18, 19]. The most commonly used biomarkers for sepsis in clinical practice are PCT and CRP. Although CRP exhibits high sensitivity, its specificity is poor, as it elevates in various inflammatory conditions. Conversely, PCT demonstrates good specificity for bacterial infections, but its accuracy in assessing disease severity and prognosis is limited [20, 21]. There is an urgent clinical need for novel biomarkers to assist in the diagnosis and management of sepsis.

IL1R2 is the decoy receptor for interleukin-1 (IL-1α/β) [12]. It was first discovered to inhibit excessive inflammatory responses by binding IL-1 and thereby preventing its activation of the pro-inflammatory signaling pathway IL1R1 [12]. IL1R2 plays a significant role in multiple diseases. For instance, its overexpression in breast and colorectal cancers induces an immunosuppressive microenvironment that promotes tumour growth [22]. Conversely, reduced IL1R2 expression in inflammatory bowel disease increases susceptibility and leads to disease progression [23]. Recent studies reveal its critical role in sepsis: IL1R2 binds to enolase 1 (ENO1) to inhibit glycolysis, thereby reducing pyroptosis and inflammation [12]. Reports indicate that IL1R2 on monocyte membranes is upregulated in sepsis but downregulated in septic shock, correlating positively with SOFA scores and serving as a marker for sepsis severity [15]. However, its detection via flow cytometry and non-linear relationship with disease severity makes clinical application inconvenient. Consequently, we explored the potential of serum IL1R2 as a biomarker for sepsis.

In this study, by comparing serum IL1R2 levels within 24 h of admission between sepsis and non-sepsis patients, we found that serum IL1R2 levels were significantly higher in sepsis patients than in non-sepsis patients [64.6 (35.3, 133.1 vs 27.0 (23.8, 35.7), *p* < 0.001]. Furthermore, serum IL1R2 levels in infected patients were also higher than in non-infected patients [25.3 (22.5, 25.7) vs 33.7(26.6, 52.4), *p* = 0.198], though this did not reach statistical significance, potentially due to the small number of patients enrolled. This suggests that IL1R2 is elevated in the context of infection, particularly during sepsis.

In terms of diagnostic efficacy for sepsis, serum IL-1R2 demonstrates excellent diagnostic value, with an AUC reaching 0.908, which is slightly lower than PCT (AUC = 0.928) but superior to CRP (AUC = 0.874). Moreover, its specificity (0.89) and positive predictive value (0.85) both exceed those of PCT and CRP, indicating that I1LR2 may be more suitable for confirmatory testing than PCT and CRP. This makes up for the shortcoming of PCT having a low positive predictive value in the diagnosis of sepsis [24], making it suitable for diagnostic confirmation in high-risk populations.

In terms of stratifying sepsis severity, patients with elevated serum IL-1R2 levels exhibited higher SOFA scores, 28-day mortality rates, incidence of shock, and mechanical ventilation requirements. Concurrently, we noted that patients with elevated serum IL1R2 levels exhibited a significantly higher proportion of G-bacterial infections, consistent with previous reports [25]. Laboratory parameters such as IL-6, lactate, ALT, PT, and PLT were also worse, indicating more severe disease. The SOFA score represents a standard for assessing the extent of organ involvement in sepsis [26]. However, its inclusion of numerous parameters renders it not convenient for clinical use [27]. Correlation analysis demonstrated a significant positive association between serum IL1R2 levels and SOFA scores, indicating strong predictive capability for disease severity. In contrast, PCT showed weaker correlation with SOFA, while CRP exhibited no correlation. Serum IL1R2 levels were also significantly elevated in patients with shock and mechanical ventilation. PCT demonstrated significant differences only in the shock group, whereas CRP showed no variation across either classification. These findings suggest that serum IL1R2 possesses superior risk stratification capabilities compared to currently established biomarkers.

Regarding the prognosis of sepsis, the predictive value of the existing biomarker PCT (particularly at admission) remains controversial [28]. Current evidence indicates that a single PCT measurement has limited value and does not support relying solely on admission PCT levels to guide clinical decision-making [4]. Instead, dynamic monitoring is more crucial [3]. Our KM analysis revealed that patients with elevated serum IL1R2 levels within 24 h of admission exhibited significantly higher 28-day mortality rates, with a negative correlation observed between IL1R2 levels and survival duration. The AUC for prognostic assessment reached 0.72. Conversely, PCT and CRP levels at admission failed to accurately predict prognosis. This suggests serum IL1R2 may serve as a prognostic indicator for sepsis.

Functionally, IL1R2 primarily acts as a decoy receptor, binding and neutralizing proinflammatory cytokines IL-1α and IL-1β to inhibit downstream inflammatory signaling. This function is critical in sepsis, as excessive IL-1 secretion is a key driver of the inflammatory storm and tissue damage associated with the condition. Concurrently, IL1R2 reduces macrophage glycolysis by inhibiting enolase 1 (ENO1) and suppresses pyroptosis and inflammatory cytokine release mediated by gasdermin D (GSDMD) [12]. These functions collectively suggest an anti-inflammatory role for IL1R2. Indeed, IL1R2 is expressed on circulating monocytes and correlates with immunosuppressive features such as low HLA-DR expression and high levels of immune checkpoint molecules [15]. Thus, IL1R2 serves as a negative feedback mechanism against hyperinflammatory states, reflecting the body's efforts to maintain immune homeostasis. Consequently, IL1R2 levels can indicate the severity of sepsis and serve as a marker of host immune status.

Finally, we investigated the pattern of elevated serum IL1R2 following sepsis onset using a mouse CLP model, discovering that serum IL1R2 levels significantly increased six hours post-CLP surgery. Serum IL1R2 levels begin to rise significantly 6 h after CLP surgery and continue to increase over the next 24 h. The levels return to normal around day 3 and continue to decline below normal by day 7. Recent studies indicate that circulating IL1R2 in the septic state originates from pyroptotic macrophages [12]. Theoretically, the more pronounced macrophage pyroptosis, the greater the release of IL1R2 into the circulation. Consequently, IL1R2 levels rise markedly during the early hyperinflammatory phase of the disease course. In the later stages of sepsis, as inflammation resolves, IL1R2 release decreases significantly and may even fall below normal levels could potentially reflect immune cell exhaustion. These speculations warrant further validation.

This study has limitations. First and foremost, this study is a single-center retrospective analysis with a limited sample size. This design limits the statistical power of the findings and may lead to imprecise or even inflated estimates of correlations and diagnostic performance. Furthermore, the single-center design inevitably introduces a risk of selection bias. Specifically, patients with sepsis included in this study had overall higher severity, and the generalizability of these results to external populations (e.g., different geographic regions, pathogen spectra and severity) requires further validation. Second, Baseline differences existed between sepsis and non-sepsis groups. Although current evidence does not indicate that gender or malignancy affects serum IL1R2 levels, given the upregulation of IL1R2 observed in various tumor tissues, we cannot rule out the possibility that locally elevated IL1R2 expression in tumor tissues may influence serum IL1R2 levels when tumor patients develop sepsis. Last, there remains a significant gap before it can be applied in clinical practice, including the development and standardization of a commercially available assay, the determination of context-specific cut-off values in large, and rigorous cost-effectiveness analyses.

## Conclusion

In summary, serum IL-1R2 represents a promising biomarker for sepsis, demonstrating utility not only for sepsis diagnosis but more importantly, for assessing disease severity and predicting mortality. Further prospective studies are justified to translate these findings into clinical practice.

## Data Availability

The data sets generated during and/or analyzed during the current study are availablefrom the corresponding author upon reasonable request.

## References

[1] Singer M, Deutschman CS, Seymour CW, Shankar-Hari M, Annane D, Bauer M, et al. The third international consensus definitions for sepsis and septic shock (Sepsis-3). JAMA. 2016;315(8):801–10. 26903338 10.1001/jama.2016.0287 PMC4968574

[2] Rudd KE, Johnson SC, Agesa KM, Shackelford KA, Tsoi D, Kievlan DR, et al. Global, regional, and national sepsis incidence and mortality, 1990-2017: analysis for the global burden of disease study. Lancet (London, England). 2020;395(10219):200–11. 31954465 10.1016/S0140-6736(19)32989-7 PMC6970225

[3] Póvoa P, Coelho L, Dal-Pizzol F, Ferrer R, Huttner A, Conway Morris A, et al. How to use biomarkers of infection or sepsis at the bedside: guide to clinicians. Intensive Care Med. 2023;49(2):142–53. 36592205 10.1007/s00134-022-06956-y PMC9807102

[4] Maves RC, Enwezor CH. Uses of procalcitonin as a biomarker in critical care medicine. Infect Dis Clin North Am. 2022;36(4):897–909. 36328642 10.1016/j.idc.2022.07.004

[5] Pierrakos C, Velissaris D, Bisdorff M, Marshall JC, Vincent JL. Biomarkers of sepsis: time for a reappraisal. Crit Care. 2020;24(1):287. 32503670 10.1186/s13054-020-02993-5 PMC7273821

[6] Zhukova JV, Lopatnikova JA, Alshevskaya AA, Sennikov SV. Molecular mechanisms of regulation of IL-1 and its receptors. Cytokine Growth Factor Rev. 2024;80:59–71. 39414547 10.1016/j.cytogfr.2024.09.004

[7] Supino D, Minute L, Mariancini A, Riva F, Magrini E, Garlanda C. Negative regulation of the IL-1 system by IL-1R2 and IL-1R8: relevance in pathophysiology and disease. Front Immunol. 2022;13:804641. 35211118 10.3389/fimmu.2022.804641 PMC8861086

[8] Molgora M, Supino D, Mantovani A, Garlanda C. Tuning inflammation and immunity by the negative regulators IL-1R2 and IL-1R8. Immunol Rev. 2018;281(1):233–47. 29247989 10.1111/imr.12609 PMC5922415

[9] Peters VA, Joesting JJ, Freund GG. IL-1 receptor 2 (IL-1R2) and its role in immune regulation. Brain Behav Immun. 2013;32:1–8. 23195532 10.1016/j.bbi.2012.11.006 PMC3610842

[10] Pyrillou K, Humphry M, Kitt LA, Rodgers A, Nus M, Bennett MR, et al. Loss of T follicular regulatory cell-derived IL-1R2 augments germinal center reactions via increased IL-1. JCI Insight. 2024;9:5. 10.1172/jci.insight.174005 PMC11143922 38329807

[11] Zhang L, Qiang J, Yang X, Wang D, Rehman AU, He X, et al. IL1R2 blockade suppresses breast tumorigenesis and progression by impairing USP15-dependent BMI1 stability. Adv Sci. 2020;7(1):1901728. 10.1002/advs.201901728 PMC6947699 31921558

[12] Tan C, Ma H, Chen J, Ma G, Jha A, Tan S, et al. Critical role of IL1R2-ENO1 interaction in inhibiting glycolysis-mediated pyroptosis for protection against lethal sepsis. Adv Sci. 2025. 10.1002/advs.202502297. 10.1002/advs.202502297 PMC12533375 40704655

[13] Guo X, Zhang Y, Zheng L, Zheng C, Song J, Zhang Q, et al. Global characterization of T cells in non-small-cell lung cancer by single-cell sequencing. Nat Med. 2018;24(7):978–85. 29942094 10.1038/s41591-018-0045-3

[14] Eum HH, Kwon M, Ryu D, Jo A, Chung W, Kim N, et al. Tumor-promoting macrophages prevail in malignant ascites of advanced gastric cancer. Exp Mol Med. 2020;52(12):1976–88. 33277616 10.1038/s12276-020-00538-y PMC8080575

[15] Supino D, Davoudian S, Silva-Gomes R, Piovani D, Garuti R, Desai A, et al. Monocyte-macrophage membrane expression of IL-1R2 is a severity biomarker in sepsis. Cell Death Dis. 2025;16(1):269. 40204720 10.1038/s41419-025-07597-x PMC11982311

[16] Gong W, Wen H. Sepsis induced by cecal ligation and puncture. Methods Mol Biol. 2019;1960:249–55. 30798538 10.1007/978-1-4939-9167-9_22

[17] Tanak AS, Sardesai A, Muthukumar S, Prasad S. Simultaneous detection of sepsis host response biomarkers in whole blood using electrochemical biosensor. Bioeng Transl Med. 2022;7(3):e10310. 36176597 10.1002/btm2.10310 PMC9471994

[18] Yan H, Zhang Y, Shi Y, Ding J, Su H, Su W, et al. Combining CD64 and CD123 biomarkers for sepsis early diagnosis and severity assessment via PD-L1 antibody affinity microfluidic (PAAM) chip in trace clinical samples. Anal Chem. 2025;97(14):7928–37. 40177943 10.1021/acs.analchem.4c07123

[19] Kellum JA, Artigas A, Gunnerson KJ, Honore PM, Kampf JP, Kwan T, et al. Use of biomarkers to identify acute kidney injury to help detect sepsis in patients with infection. Crit Care Med. 2021;49(4):e360–8. 33566467 10.1097/CCM.0000000000004845 PMC7963439

[20] Zaki HA, Bensliman S, Bashir K, Iftikhar H, Fayed MH, Salem W, et al. Accuracy of procalcitonin for diagnosing sepsis in adult patients admitted to the emergency department: a systematic review and meta-analysis. Syst Rev. 2024;13(1):37. 38254218 10.1186/s13643-023-02432-w PMC10802075

[21] Song J, Park DW, Moon S, Cho HJ, Park JH, Seok H, et al. Diagnostic and prognostic value of interleukin-6, pentraxin 3, and procalcitonin levels among sepsis and septic shock patients: a prospective controlled study according to the Sepsis-3 definitions. BMC Infect Dis. 2019;19(1):968. 31718563 10.1186/s12879-019-4618-7 PMC6852730

[22] Arnouk SM, Kancheva D, Van Damme H, Courtoy GE, Mora Barthelmess R, Van Craenenbroeck J, et al. Depleting IL1R2+ tumor-infiltrating regulatory T cells with an ADCC-prone nanobody construct boosts the efficacy of anti-PD1 immunotherapy. Cancer Res. 2025. 10.1158/0008-5472.can-24-3095. 40960523 10.1158/0008-5472.CAN-24-3095 PMC12666320

[23] Mora-Buch R, Dotti I, Planell N, Calderón-Gómez E, Jung P, Masamunt MC, et al. Epithelial IL-1R2 acts as a homeostatic regulator during remission of ulcerative colitis. Mucosal Immunol. 2016;9(4):950–9. 26530134 10.1038/mi.2015.108 PMC4917674

[24] Bolanaki M, Winning J, Slagman A, Lehmann T, Kiehntopf M, Stacke A, et al. Biomarkers improve diagnostics of sepsis in adult patients with suspected organ dysfunction based on the quick sepsis-related organ failure assessment (qSOFA) score in the emergency department. Crit Care Med. 2024;52(6):887–99. 38502804 10.1097/CCM.0000000000006216 PMC11093432

[25] Lang Y, Jiang Y, Gao M, Wang W, Wang N, Wang K, et al. Interleukin-1 receptor 2: a new biomarker for sepsis diagnosis and gram-negative/gram-positive bacterial differentiation. Shock (Augusta, Ga). 2017;47(1):119–24. 27984536 10.1097/SHK.0000000000000714

[26] Alrawashdeh M, Klompas M, Rhee C. The impact of common variations in sequential organ failure assessment score calculation on sepsis measurement using Sepsis-3 criteria: a retrospective analysis using electronic health record data. Crit Care Med. 2024;52(9):1380–90. 38780372 10.1097/CCM.0000000000006338

[27] Martin-Loeches I, Singer M, Leone M. Sepsis: key insights, future directions, and immediate goals. A review and expert opinion. Intensive Care Med. 2024;50(12):2043–9. 39531053 10.1007/s00134-024-07694-z

[28] Lawandi A, Oshiro M, Warner S, Diao G, Strich JR, Babiker A, et al. Reliability of admission procalcitonin testing for capturing bacteremia across the sepsis spectrum: real-world utilization and performance characteristics, 65 U.S. hospitals, 2008-2017. Crit Care Med. 2023;51(11):1527–37. 37395622 10.1097/CCM.0000000000005968

